# Identification of anoikis-related genes in heart failure: bioinformatics and experimental validation

**DOI:** 10.1186/s41065-025-00532-2

**Published:** 2025-08-16

**Authors:** Lina Zhang, Jianjun Gu, Yan Jiang, Juan Xue, Ye Zhu

**Affiliations:** 1 Department of Cardiology, Affiliated Hospital of Nantong University, Nantong, Jiangsu China; 2 Department of Respiratory and Critical Care Medicine, Affiliated Hospital and Medical School of Nantong University, Nantong, China; 3 Department of Cardiology, Northern Jiangsu People’s Hospital, Yangzhou University, 98 Nantong West Road, Yangzhou, Jiangsu China; 4 Department of Obstetrics, Xuzhou Cancer Hospital, Xuzhou, 221005 Jiangsu Province China; 5 Department of Cardiology, Northern Jiangsu People’s Hospital, Yangzhou, Jiangsu, China

**Keywords:** Anoikis, Machine learning, Bioinformatics, Heart failure, Hub genes

## Abstract

**Background:**

Heart failure (HF) is a common clinical syndrome caused by ventricular dysfunction and one of the leading causes of mortality worldwide. Previous studies have suggested that anoikis is relevant to HF. This study aimed to identify hub genes associated with anoikis that may offer therapeutic targets for HF.

**Materials and methods:**

Gene expression data for GSE36074 were obtained from the Gene Expression Omnibus (GEO) and anoikis-related genes (ARGs) were extracted from GeneCards. GEO2R was used to screen for differentially expressed genes (DEGs), then by overlapping DEGs with ARGs, differentially expressed ARGs (DEARGs) were screened. The biological functions of the DEARGs were determined using DAVID. Subsequently, two machine learning (ML) algorithms were employed to identify hub DEARGs: least absolute shrinkage and selection operator (LASSO) and random forest (RF). In addition, miRNA-hub DEARGs and drug-hub DEARGs networks were constructed. Lastly, the hub DEARGs were validated by quantitative reverse transcription PCR (RT-qPCR) and Immunofluorescence (IF).

**Results:**

A total of 138 DEARGs were identified in GSE36074. Functional analysis of DEARGs revealed that they were primarily enriched in the positive regulation of the apoptotic process, PI3K-Akt, and FoxO signaling pathways. Subsequently, two hub DEARGs (Tln1 and TGFβ2) were screened using LASSO and RF algorithms. According to the miRNA–hub DEARGs networks, Tln1 and TGFβ2 were regulated by 34 and 68 miRNAs, respectively. Moreover, drug-hub DEARGs networks showed that Gemogenovatucel-t, Lerdelimumab, Belagenpumatucel-l, Fresolimumab, Bintrafusp alfa, Trabedersen and Luspatercept-aamt are potential drugs that could target TGFβ2. Finally, RT-qPCR and IF validation of two key DEARGs (Tln1 and TGFβ2) supported our bioinformatics analysis.

**Conclusions:**

These findings suggest that Tln1 and TGFβ2 may play important roles in HF development through the regulation of anoikis and may serve as therapeutic targets for HF.

**Clinical trial number:**

Not applicable.

**Supplementary Information:**

The online version contains supplementary material available at 10.1186/s41065-025-00532-2.

## Introduction

Heart failure (HF) is a common outcome of most cardiovascular diseases (CVDs) [1]. Owing to population aging and longer survival after myocardial infarction (MI), its prevalence is increasing [2]. Consequently, HF is a serious economic burden worldwide. Although HF has undergone significant improvements in treatment, it remains associated with a high mortality rate. Therefore, a deeper understanding of HF pathogenesis may lead to the identification of novel therapeutic targets.

A unique form of programmed cell death, anoikis occurs when cells detach from their extracellular matrix (ECM) [3]. Tissue homeostasis is maintained by preventing abnormal cell growth and adhesion to inappropriate ECM [4]. Studies have proven that Anoikis is involved in the progression of malignancies [5], ischemic stroke [4], and spinal cord injury [6]. Moreover, it is also worth mentioning that anoikis contribute to CVD pathogenesis. Franck et al. reported that neutrophil elastase levels were increased in patients with acute MI (AMI), which can degrade basement membrane components, exacerbate endothelial cell injury, and promote anoikis [7]. Wang et al. discovered that matrix metalloproteinases (MMPs) could degrade collagen and elastin in the ECM and may induce anoikis in aortic dissection [8]. In addition, Ding et al. observed aberrant myocyte branching and β_1_-integrin deposition in left ventricular (LV) myocytes in a mouse model of ascending aortic stenosis and confirmed that anoikis contributes significantly to the transition from adaptive hypertrophy to early HF [9]. Thus, it is essential to study the contribution of anoikis-related genes (ARGs) in HF.

Transverse aortic constriction (TAC), as a recognized experimental model for mechanical overload-induced cardiac remodeling and subsequent HF, has been widely used to elucidate fundamental signaling processes associated with myocardial hypertrophy, providing a more gradual temporal process for HF development [10]. In the current study, we first analyzed the DEARGs in HF, then performed clustering and functional enrichment analyses of the DEARGs and used two ML algorithms to identify hub characteristic genes. Next, gene-miRNA and gene-drug predictions of the characteristic genes were conducted. Finally, the characteristic genes were validated using an external dataset and animal experiments. The findings of this study suggest new therapeutic targets for HF.

## Methods

### Data acquisition

The mRNA expression matrices (GSE36074 and GSE24454) were downloaded from the GEO on platform GPL1261([Mouse430_2] Affymetrix Mouse Genome 430 2.0 Array). To explore early pathology of heart failure under mechanical stress, the murine TAC datasets(After four weeks of TAC)were included. The dataset with less than 3 samples per group is excluded. The GSE36074 dataset (five Sham and Seven TAC samples) was used for subsequent analyses. The GSE24454 dataset (three Sham and three TAC samples) was used as the validation cohort. ARGs were extracted from the GeneCard database.

### Identification of differentially expressed ARGs (DEARGs)

GEO2R was used to screen for DEGs. Genes with a | Fold change) | ≥1 and adj.*p* < 0.05 were considered DEGs. Using the Draw Venn Diagram tool, DEARGs were generated from DEGs and ARGs.

### Enrichment analyses of DEARGs

Gene Ontology (GO) and Kyoto Encyclopedia of Genes and Genomes (KEGG) analyses of the DEARGs were performed using DAVID 6.8. Significantly enriched pathways were defined as those with adj.*p* < 0.05.

### Identification of hub DEARGs

To further screen for hub DEARGs, two ML algorithms were employed. LASSO regression is a commonly used model for classification problems, and can be used for feature selection [11]. LASSO was conducted using the R glmnet package with 5-fold cross-validation [12]. The RF algorithm is an ensemble learning method that uses multiple decision trees to classify the significant genes [13]. The RF analysis was performed using the R random forest package. Genes identified by both LASSO and RF were identified as hub DEARGs. To evaluate the accuracy of the hub DEARGs, the “pROC” package was utilized to conduct a receiver operating characteristic (ROC) curve analysis.

### Regulatory networks of miRNA-DEARGs

MiRNAs play critical regulatory roles at the mRNA level. Thus, the upstream miRNAs for the hub DEARGs were predicted using the miRWalk 3.0. To increase the accuracy of the predictions, both the miRDB and miRWalk databases were used to screen for miRNAs. The miRNA-DEARG networks were visualized using Cytoscape.

### Predicting target drugs of hub DEARGs

To identify therapeutic drugs that targeted key DEARGs, the DGIdb database was employed [14]. The drugs-DEARGs network was visualized using Cytoscape.

### Mouse transverse aortic constriction (TAC) model construction

The mouse TAC model is commonly used to simulate human CVD and study the underlying mechanisms of HF. Male SPF C57BL/6 mice (aged 8–10 weeks) were purchased from Yangzhou University and housed under standard conditions. After acclimatization for 7 days, the mice were randomly allocated to the sham and TAC groups of five mice each. TAC surgery was performed as previously described [15]. Mice in the sham group underwent a comparable procedure without aortic ligation. Subsequently, 28 days after TAC, the cardiac function was evaluated using echocardiography. All mice were anesthetized with ketamine (80 mg/kg) and xylazine (5 mg/kg) by intraperitoneal injection and euthanized with cervical dislocation, then, the hearts were collected for subsequent examination. All animal protocols were approved by the Animal Ethics Committee of Yangzhou University. The institution is accredited by the Association for Assessment and Accreditation of Laboratory Animal Care International. All experiments were performed in accordance with the Guide for the Care and Use of Laboratory Animals published by the National Institutes of Health, and ARRIVE guidelines.

### Quantitative reverse transcription PCR (RT-qPCR)

The hub DEARGs mRNA levels were determined by RT-qPCR. Briefly, total RNA was extracted from heart tissue using an RNA extraction reagent (Vazyme, China), and RNA was used to synthesize cDNA using a reverse transcription kit (Servicebio, China). The PCR primer sequences are listed in Table 1. To normalize mRNA levels, GAPDH was used as an internal reference.

**Table 1:** Pairs of forward-reverse primers

Gene	Forward		Reverse
Tln1 TGFβ2 GAPDH	TAGAGGCAACCACAGAGCACATAC GTTACAACACCCTCTGGCTCATT CCTCGTCCCGTAGACAAAATG		CATAGTAATACCCTTGGTCATTCGG GCGGACGATTCTGAAGTAGGG TGAGGTCAATGAAGGGGTCGT

### Immunofluorescent staining

Immunofluorescence analysis was performed as previously described [16]. Briefly, heart tissues were fixed with 4% paraformaldehyde solution for 1 h, permeabilized in 1.5% Triton X-100 for 30 min, and incubated in 5% FBS for 90 min. Subsequently, the samples were incubated with the primary antibody solution TLN1 (1:100; cat.no. A4158; ABclonal. Wuhan, China), and TGFβ2 (1:100; cat.no.ER1917-63; Huabio. Hangzhou, China) at 4 °C overnight. Subsequently, heart tissues were incubated with a fluorescent secondary antibody for 2 h at 37 °C. Nuclei were stained with DAPI in the dark for 15 min. Finally, heart tissues were observed under a fluorescence microscope (Olympus, Japan).

### Statistical analysis

Statistical analyses were performed using GraphPad Prism 8.0. Data are presented as mean ± standard deviation (SD). When comparing two groups, Student’s t-test (unpaired, two-tailed) was used. *p* < 0.05 was considered statistically significant.

## Results

### Screening of DEARGs

**Fig. 1:**
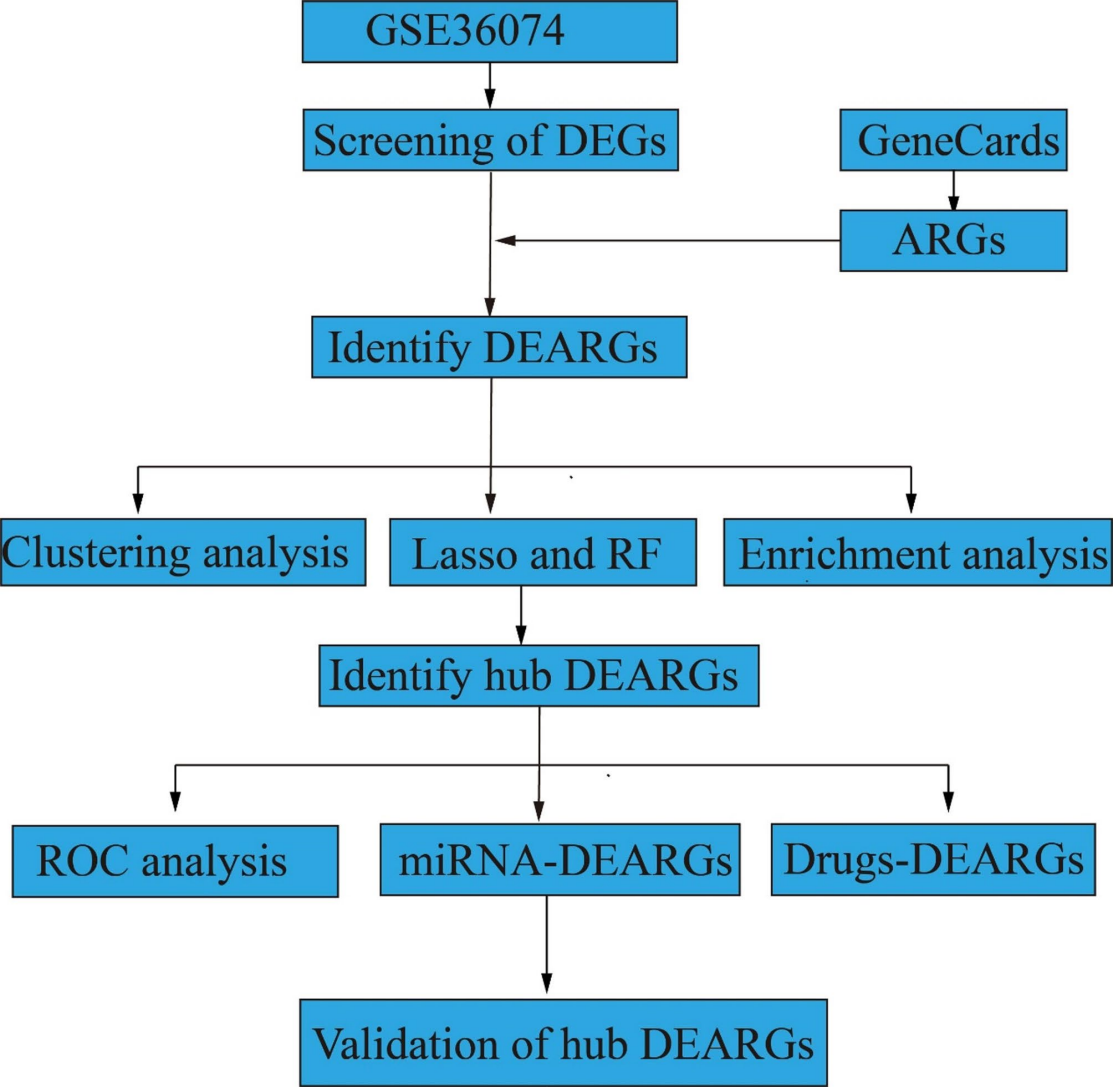
Study protocol. A flow diagram of this study protocol

**Fig. 2:**
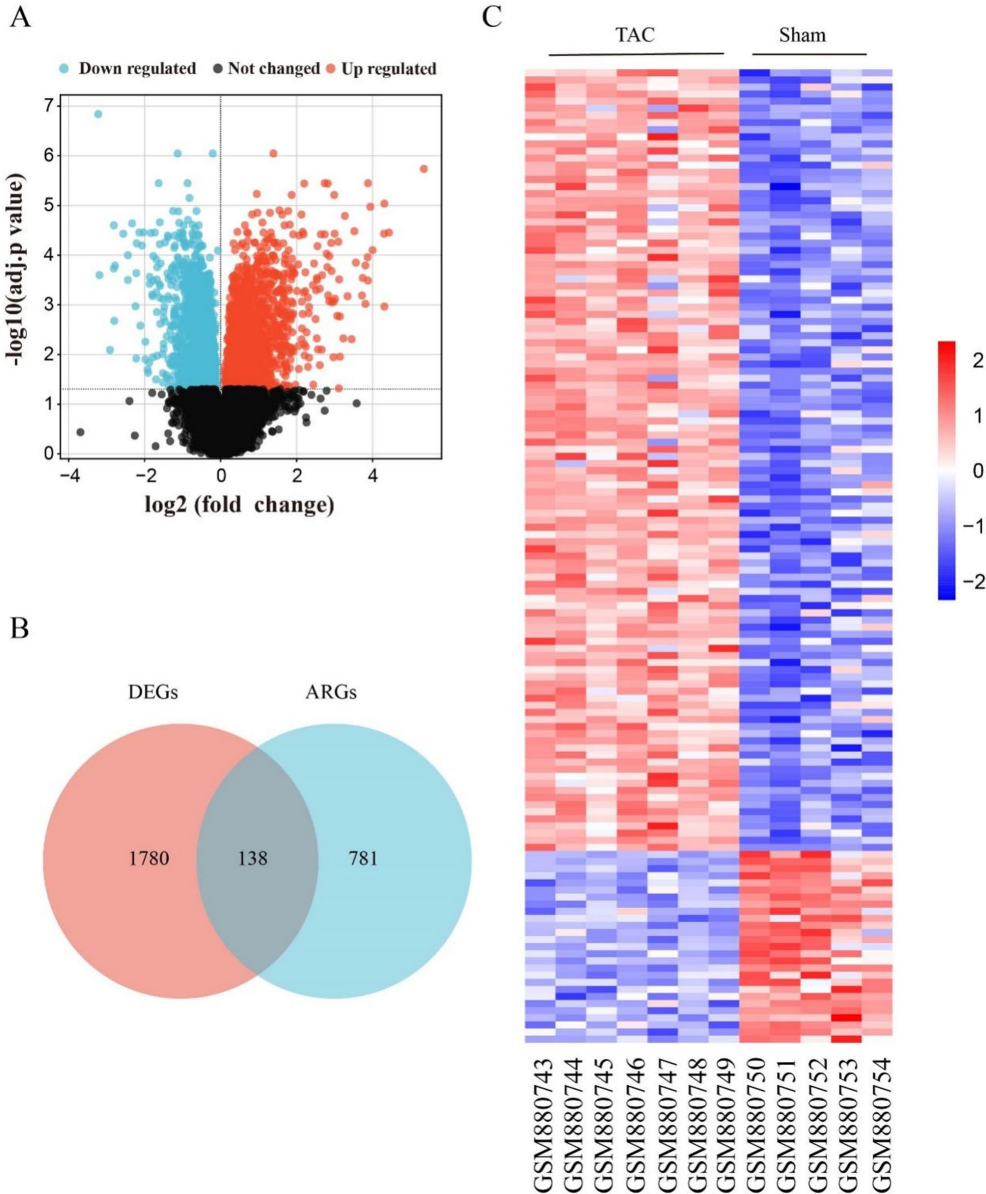
The differential expression of ARGs (DEARGs) in HF. (**A**) Differentially expressed genes (DEGs) plotted in a volcano. Genes marked in red are upregulated, while genes marked in blue are downregulated. (**B**) Venn diagrams illustrating the overlap between intersecting genes of ARGs and DEGs. (**C**) Heatmap of DEARG-based clustering analyses. ARGs: anoikis-related genes

The flow diagram of this study is shown in Fig. 1. In the GSE36074 dataset, a total of 1918 DEGs were identified (Fig. 2A), and we found 919 ARGs in the GeneCards dataset, after intersecting with DEGs, 138 DEARGs were screened (Fig. 2B). In addition, based on the clustering analysis of DEARGs, samples from the same group tended to cluster together (Fig. 2C).

### GO and KEGG enrichment analyses of DEARGs

**Fig. 3:**
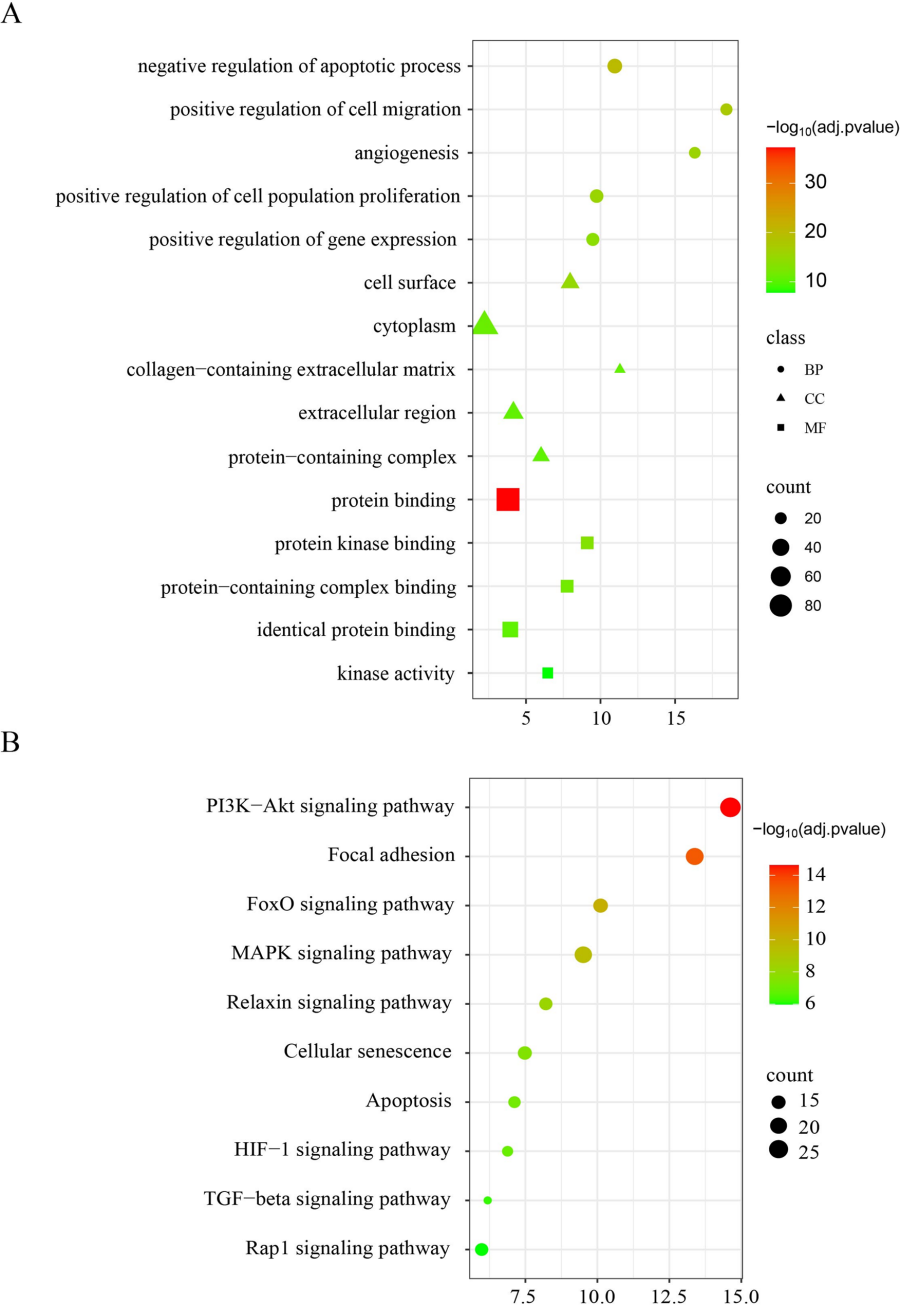
Analysis of DEARGs was conducted to identify functional enrichment. (**A**) Enrichment of DEARGs by GO. (**B**) Pathway analysis of DEARGs by KEGG. BP: biological processes; CC: cellular component. MF: molecular function

To elucidate the DEARGs, we performed GO and KEGG enrichment analyses using the DAVID database. For GO analysis, the top five biological process (BP) terms were negative regulation of the apoptotic process, positive regulation of cell migration, angiogenesis, positive regulation of cell population proliferation and gene expression; The top five cellular component (CC) were cell surface, cytoplasm, collagen-containing extracellular matrix, extracellular region, and protein-containing complex; For molecular function (MF), protein binding, protein kinase binding, protein-containing complex binding, identical protein binding, and kinase activity were most enriched (Fig. 3A). Additionally, KEGG analysis indicated that DEARGs were closely enriched in the PI3K-Akt, FoxO, MAPK, Relaxin, HIF-1, TGF-beta and Rap1 signaling pathways, focal adhesion, cellular senescence, and apoptosis (Fig. 3B).

### Screening hub DEARGs by machine learning

**Fig. 4:**
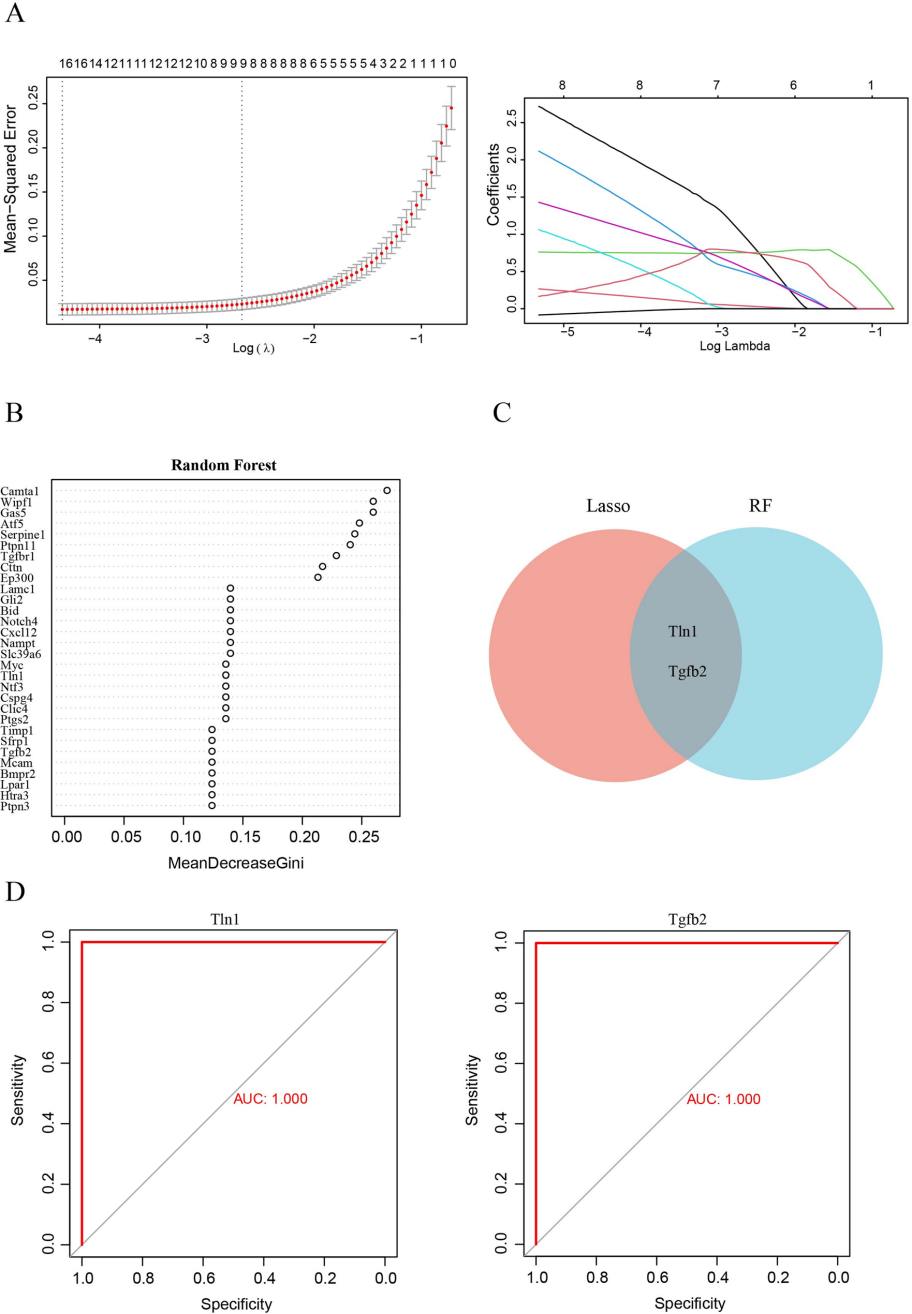
The screened key DEARGs by machine learning. (**A-B**) The LASSO and RF algorithms were used to identify key DEARGs. (**C**) Venn diagrams illustrating the overlap between intersecting genes of LASSO and RF (**D**) the ROC of Tln1 and TGFβ2

To further identify hub DEARGs related to HF, the LASSO and RF algorithms were employed. Based on LASSO analysis, 8 DEARGs were confirmed (Fig. 4A), and 33 DEARGs were screened by RF analysis (Fig. 4B). Moreover, after the intersection between the LASSO analysis and RF results, two common hub DEARGs (Tln1 and TGFβ2) were obtained (Fig. 4C).

Moreover, we calculated the AUC-ROC value for each hub DEARGs, which resulted in a value of 1 for Tln1 and TGFβ2 (Fig. 4D). This indicates that hub DEARGs are highly effective for predicting HF.

### MiRNA target gene prediction and drug prediction of key gene

**Fig. 5:**
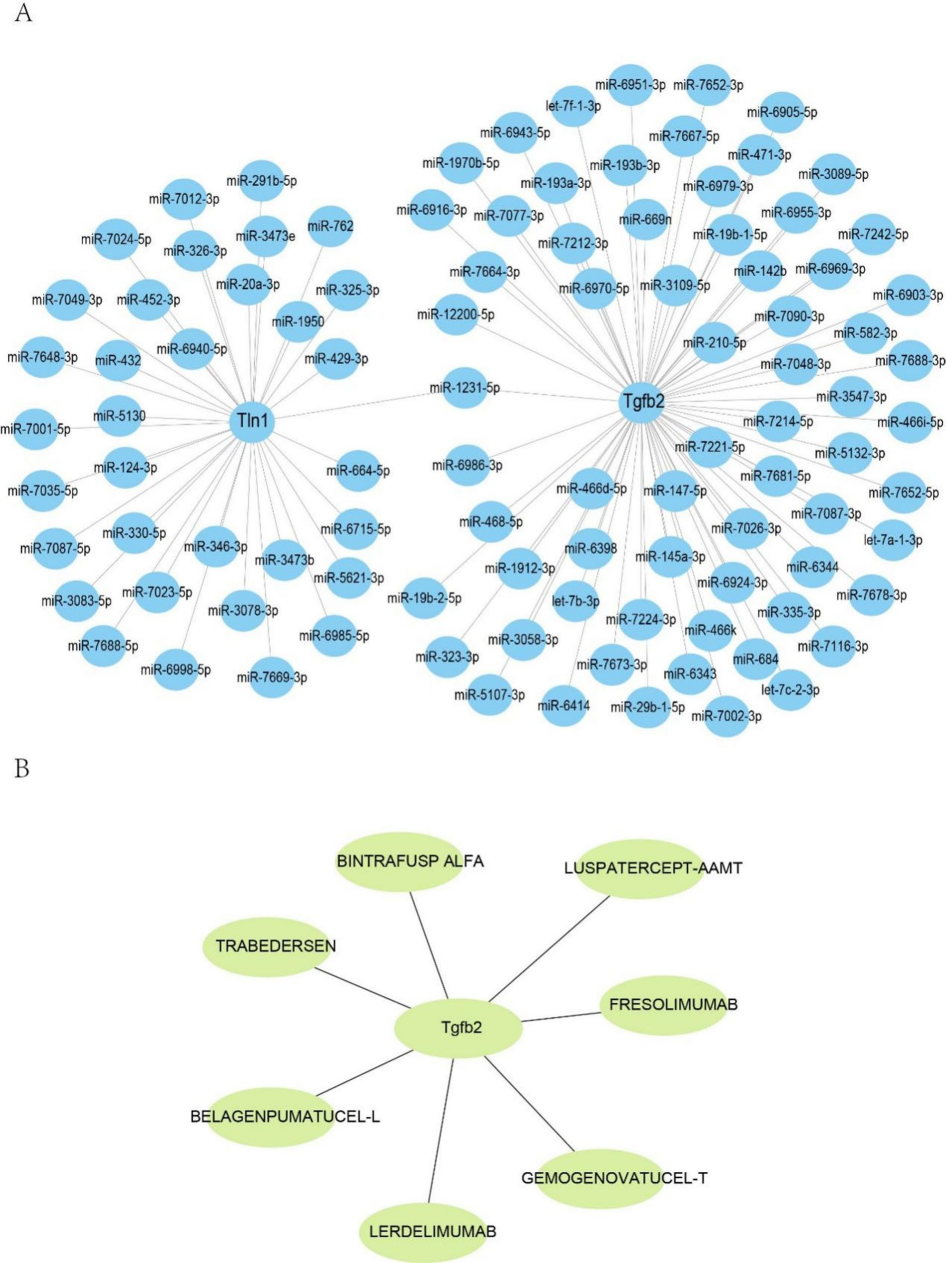
Construction of miRNA-DEARGs and Drug-DEARGs regulatory network. (**A**) miRNA-DEARGs network construction. (**B**) Drug prediction of Tln1 and TGFβ2 by using DGIdb database

In post-transcriptional regulation, miRNAs play a critical role in gene expression. In this study, to identify potential miRNA targets of the hub DEARGs, we used the miRWalk database. Based on miRNA-gene networks, we discovered that Tln1 and TGFβ2 were regulated by 34 and 68 miRNAs, respectively. Furthermore, miR-1231-5p co-regulated TGFβ2 and Tln1 expression (Fig. 5A). In addition, to investigate therapeutic drugs that could target hub DEARGs, DGIdb database was employed. The results showed that Gemogenovatucel-t, Lerdelimumab, Belagenpumatucel-l, Fresolimumab, Bintrafusp alfa, Trabedersen and Luspatercept-aamt are potential drugs that could target TGFβ2(Fig. 5B). However, no therapeutic drugs targeting Tln1 have been identified.

### Validation of hub DEARGs

**Fig. 6:**
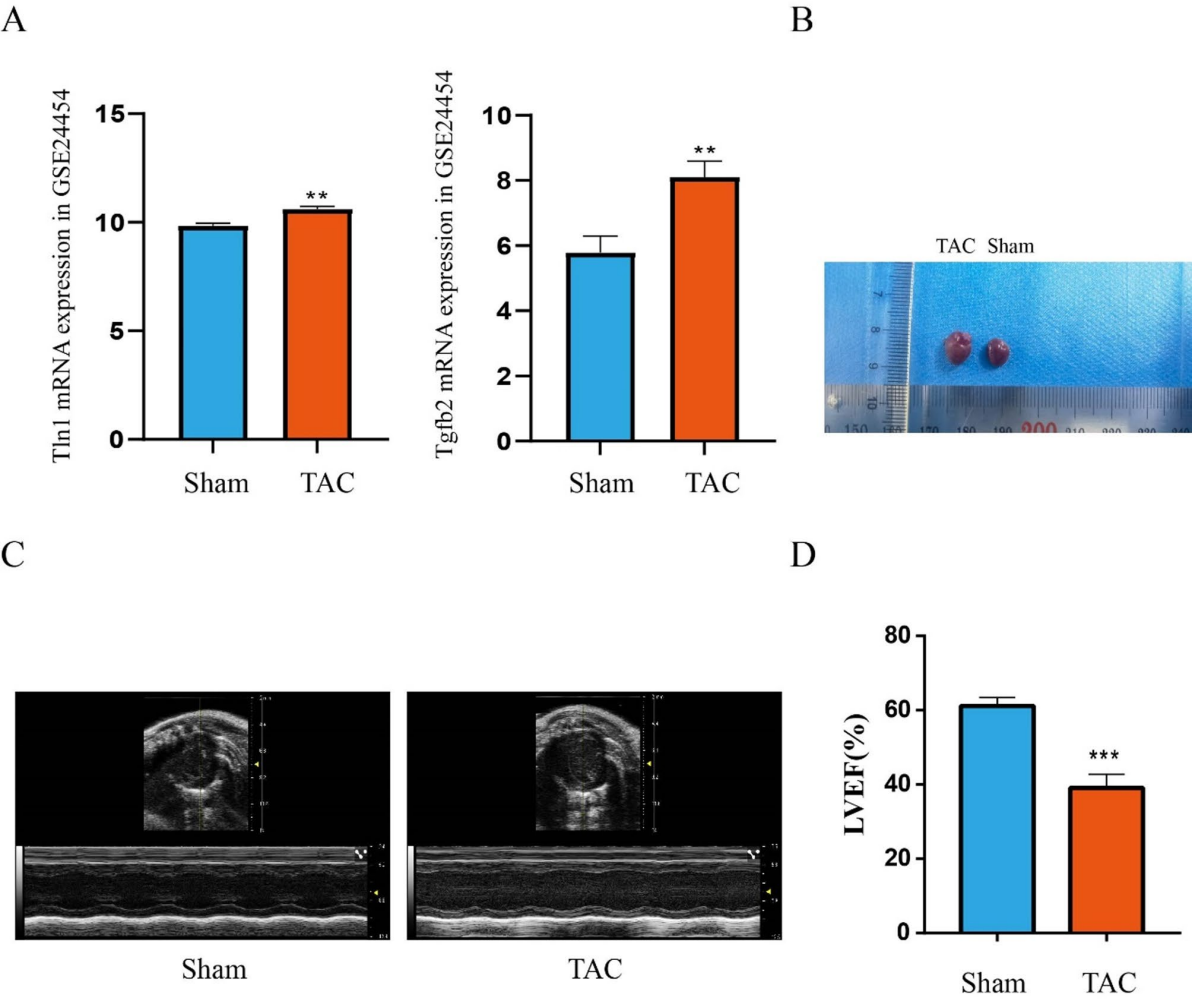
An established model for mouse HF induced by TAC. (**A**) the mRNA expression levels of Tln1 and TGFβ2 in GSE24454 dataset. (**B**) The heart size of Sham and TAC mice. (**C**) Images of heart ultrasound. (**D**). LVEF: left ventricular ejection fraction. Statistical values are given as mean (± SD) (n=5). **p < 0.01 and *** *p* < 0.001vs Sham group

To validate the two hub DEARGs levels, their mRNA expression profiles were detected using the GSE24454 dataset. There was a significant increase in Tln1 and TGFβ2 expression in the TAC group compared to the sham group, suggesting they may be involved in HF (Fig. 6A). We also validated Tln1 and TGFβ2 using human patient heart failure datasets(GSE116250). Compared to the Control group, the expression of Tln1 and TGFβ2 was significantly increased in the human patient heart failure group (SFigure 1). For further the validation of hub DEARG expression, a mouse TAC model was developed. After 4 weeks of TAC, we found that the heart size of TAC mice was significantly larger than those of sham mice (Fig. 6B); Furthermore, echocardiography was performed on mice, and the results showed that the TAC group had significantly lower left ventricular ejection fraction (LVEF) than the sham group (Fig. 6C-D); A TAC-induced HF model has been successfully established in mice based on those results. Meanwhile, we confirmed that anoikis does occur in the early stages of heart failure through immunofluorescence and TUNEL staining (SFigure 2 A-B).

**Fig. 7:**
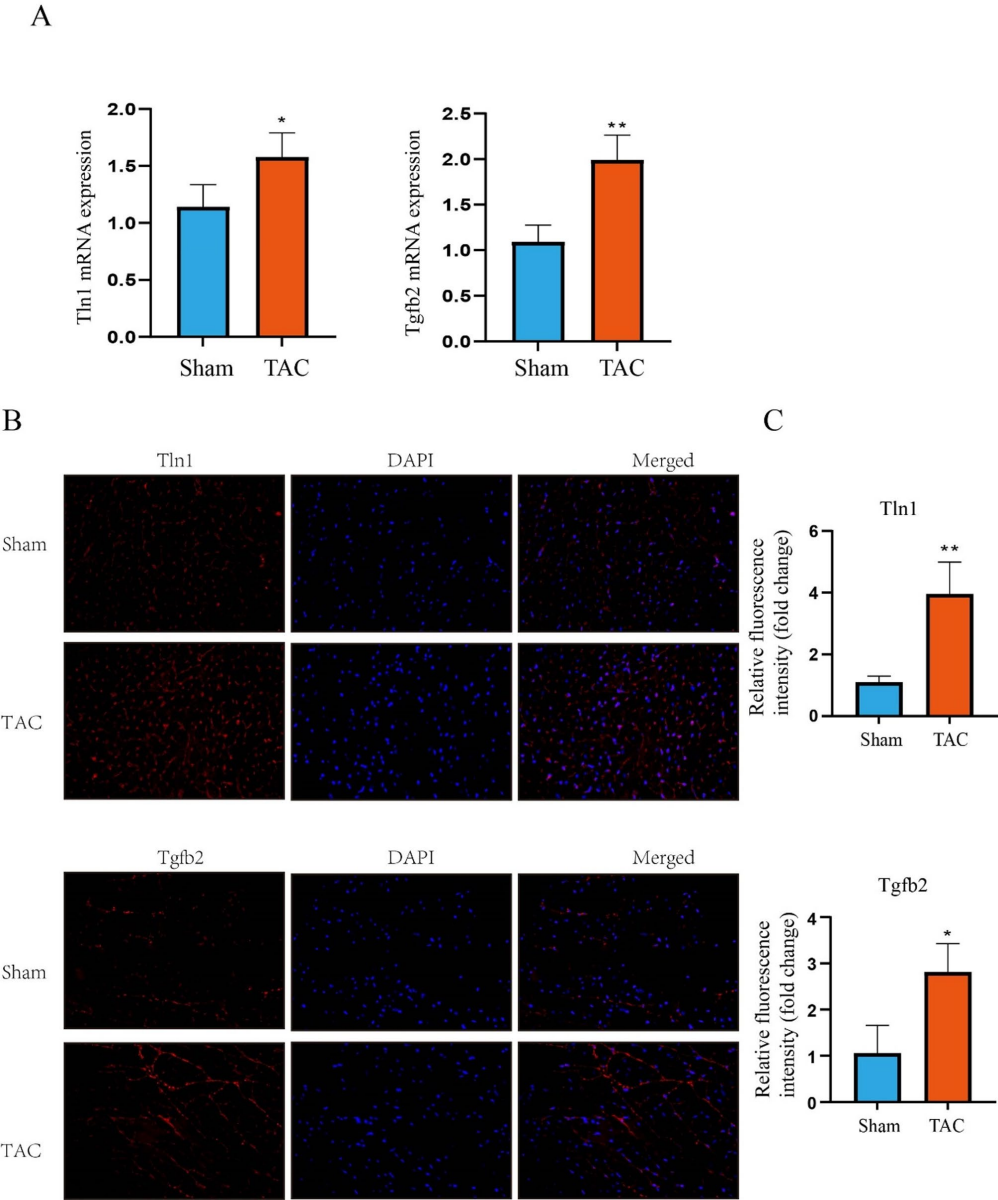
Verification of key DEARGs expression at both mRNA and protein levels. (**A**)Tln1 and TGFβ2 mRNA level in the TAC and Sham groups. Expression levels were standardized for GAPDH levels, Statistical values are given as mean (± SD) (*n* = 4). (**B**) Immunofluorescence (IF) staining of Tln1 and TGFβ2 proteins in the TAC and Sham groups. (**C**) Quantification of immunofluorescence intensity of Tln1 and TGFβ2. Statistical values are given as mean (± SD) (*n* = 3), *p < 0.05 and **p < 0.01 vs. Sham group. Scale bar: 50 μm

RT-qPCR analysis revealed that Tln1 and TGFβ2 mRNA levels were significantly higher in the TAC group than in the sham group (Fig. 7A). Furthermore, these hub genes were further verified at the protein level using IF (Fig. 7B-C). It was observed that the protein levels of Tln1 and TGFβ2 in the TAC group were significantly higher than those in the sham group (*p* < 0.05). The results of these analyses were consistent with those of the bioinformatics analysis.

## Discussion

HF is a chronic disease and its pathogenesis is multifactorial, leading to high morbidity and mortality. Previous research has shown that anoikis contributes significantly to the development and progression of CVDs (such as AMI and aortic dissection). However, to our knowledge, no studies have employed bioinformatic analysis to connect HF with anoikis. Hence, in the current study, we performed bioinformatic analysis to explore anoikis-related hub genes associated with HF and validated it using an external dataset and animal experiments. This study provides some crucial biomarkers for the diagnosis and therapy evaluation of HF. Revealing specific gene expression patterns and exploring the underlying mechanisms behind anoikis can provide a foundation for designing and implementing novel therapeutic approaches for heart failure.

In this study, we identified 138 DEARGs by analyzing TAC and Sham group heart samples. Moreover, according to functional enrichment analysis, these DEARGs are primarily implicated in the negative regulation of the apoptotic process, PI3K-Akt, FoxO signaling pathway, and focal adhesion. It has been confirmed that patients with HF have higher levels of FoxO [17], and in mice, FoxO upregulation can lead to adverse cardiac remodeling [18]. Additionally, there is evidence that PI3K/AKT signaling protects cardiac compensatory hypertrophic responses, even preventing apoptosis in cardiomyocytes [19]. Most importantly, studies have shown that PI3K/AKT signaling plays a role in resistance to anoikis [20], but FoxO signaling can induce anoikis [21]. Our study indicates that anoikis and anoikis resistance exist simultaneously in HF.

Subsequently, to screen potential diagnostic biomarkers for HF, two machine learning algorithms were employed. ML is a valuable tool to derive accurate biomarkers for diseases. LASSO is an effective ML method for modeling datasets with limited samples and numerous independent variables and is commonly used in studies with complex features [22–24]. In addition to its advantages in feature selection, LASSO also offers strong robustness, interpretability, and high data applicability. RF, an integrated algorithm, has demonstrated high accuracy in diagnosing diseases and predicting risks [25]. With the help of two feature selection methods, we identified only two genes (Tln1 and TGF-β2) that may serve as diagnostic markers for HF. Furthermore, the ROC curve analysis results showed an AUC value of 1, indicating that Tln1 and TGF-β2 had excellent accuracy and specificity in distinguishing HF samples from normal samples.

Tln1, one of the key focal adhesions (FAs) proteins, contributes to integrins-related pathways and modulates numerous cellular activities, such as cell-ECM interactions, cell survival, mechanosensation, and signal transduction [26–31]. Previous research had indicated that Tln1 overexpression could promote prostate cancer cell metastasis and invasion through focal adhesion signaling and anoikis resistance [32], which may be related to AKT signaling activation. Moreover, a recent study showed that the absence of Tln1 in cardiac fibroblasts enhances ventricular hypertrophy during pressure overload [33]. Importantly, Manso AM et al. found that the level of Tln1 was upregulated in human failing heart and in the TAC mouse model, and demonstrated that the specific knockout of Tln1 in cardiomyocytes showed blunted hypertrophy, less fibrosis, and improved cardiac function by inhibiting the activation of ERK1/2, p38, Akt, and glycogen synthase kinase 3 in mechanical overload mice model [34]. Ding et al. confirmed that anoikis occurs in the early stage of heart failure in a mouse TAC model, and found that β1 integrin deposition in situ [9]. As an important mechanical sensor, β1 integrin has been shown to be associated with the hypertrophic growth of cardiomyocyte [35]. Previous studies have shown that β1 integrin is essential for the normal activation of a series of signaling pathways, including ERK, p38, and Akt, in mechanical stress activation in cardiomyocytes [36]. Subsequent studies have shown that knocking out Tln1 and knocking out β1 integrin yield similar results, consistent with Tln1’s crucial role as an important mediator in β1 integrin dependent mechanical stress response [34]. Based on the above research, we speculate that Tln1 may participate in the anoikis of cardiomyocyte through the activation of related apoptotic pathways by β1 integrin.

A member of the TGF family, TGF-β2 plays an essential role in regulating cell growth, differentiation, epithelial-mesenchymal transformation, cardiac myogenesis, and cardiac morphogenesis [37–40]. Corbet et al. documented that TGFβ2- mediated lipid droplet formation contributes to anoikis resistance and cancer cell invasion [41]. In addition, Koitabashi N, J et al. found Cardiomyocyte-specific TβR2 knockdown uniquely upregulates BMP7 through TAK1 signaling, thereby preventing adaptive remodeling to mechanical overload [42]. However, the role of TGFβ2 in the development of pressure overload-induced anoikis has not well understood. There is still much to learn regarding the molecular mechanisms involved in this process.

MicroRNAs (miRNAs) are non-coding RNAs that post-translationally regulate gene expression. Previous studies have reported that several miRNAs play critical roles in HF pathogenesis. Thus, we developed–A the miRNA-mRNA networks for Tln1 and TGFβ2. Based on the miRNA-hub DEARGs network, we found that miR-1231-5p simultaneously regulated Tln1 and TGFβ2 expression. However, studies related to hub DEARGs and miR-1231-5p are limited.

Finally, using the DGIdb database, we found that Gemogenovatucel-t, Lerdelimumab, Belagenpumatucel-l, Fresolimumab, Bintrafusp alfa, Trabedersen and Luspatercept-aamt to target TGFβ2. Gemogenovatucel-t, Belagenpumatucel-l, Fresolimumab and Trabedersen are TGF-β pathway inhibitors that have reached phase II clinical development in several cancers [43]. Previous research has shown that lerdelimumab (also named CAT-152), an anti-TGF beta 2 antibody, was used to prevent fibrosis progression in patients undergoing their first trabeculectomy for primary open-angle; however, a phase III study found no difference between CAT-152 and placebo in preventing primary trabeculectomy failure [44]. Bintrafusp alfa, a bifunctional fusion protein targeting TGF-β and PD-L1, showed manageable safety and efficacy for pretreated non-small cell lung cancer and esophageal squamous cell carcinoma [45, 46]. Luspatercept has been approved for β-thalassemia treatment and has shown good efficacy and effectiveness [47].

However, this study had some limitations. First, the sample size for each group was relatively small. In addition, because of the constantly updated GeneCard database, our study did not include all anoikis-related genes. Finally, we only preliminarily discovered genes related to anoikis that may be associated with heart failure through bioinformatics, and did not conduct functional validation or in-depth mechanism exploration. In the future, we will knock out genes for phenotype functional validation, perform sequencing to find reliable related pathways, and conduct *in vivo and in vitro* validation.

## Conclusion

Consequently, our study indicated that these two hub DEARGs (Tln1 and TGFβ2) are closely linked to anoikis in HF and may serve as potential targets for this disease.

## Supplementary Information

Below is the link to the electronic supplementary material.

Supplementary Material 1

## Data Availability

No datasets were generated or analysed during the current study.

## Abbreviations

ARGs: Anoikis-related genes
IF: Immunofluorescence
LASSO: least absolute shrinkage and selection operator
RF: Random forest
HF: Heart failure
