# Supplementary material for: Identification of anoikis-related genes in heart failure: bioinformatics and experimental validation
Source: Hereditas. 2025 Aug 16;162:163. doi: 10.1186/s41065-025-00532-2 (PMC12357471; doi:10.1186/s41065-025-00532-2)
Supplement: Supplementary file 1 — Supplementary Material 1 [file 41065_2025_532_MOESM1_ESM.docx]

**Supplementary materials 1**

**SFigure 1. Verification of key DEARGs expression in human patient heart failure datasets**


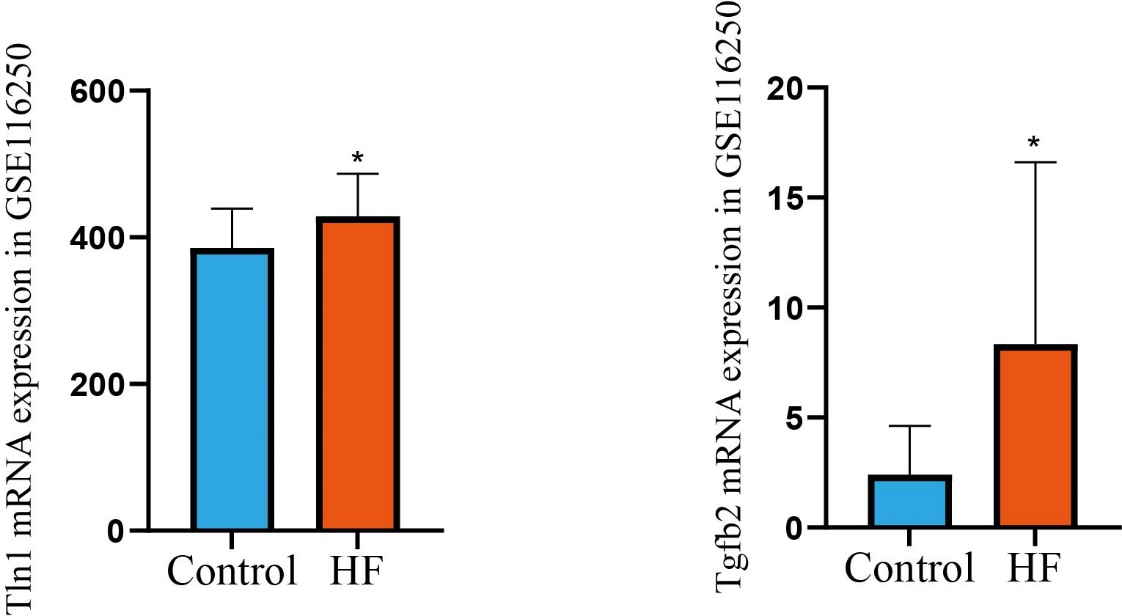


The GSE116250 dataset (14 non-failing donors and 50 patients with heart failure (HF)|). Statistical values are given as mean (± SD). **p* < 0.05 vs HF.

**SFigure 2. Up-Regulation of the β1 integrin and increased apoptosis are associated with mechanical overload induced HF in TAC model**


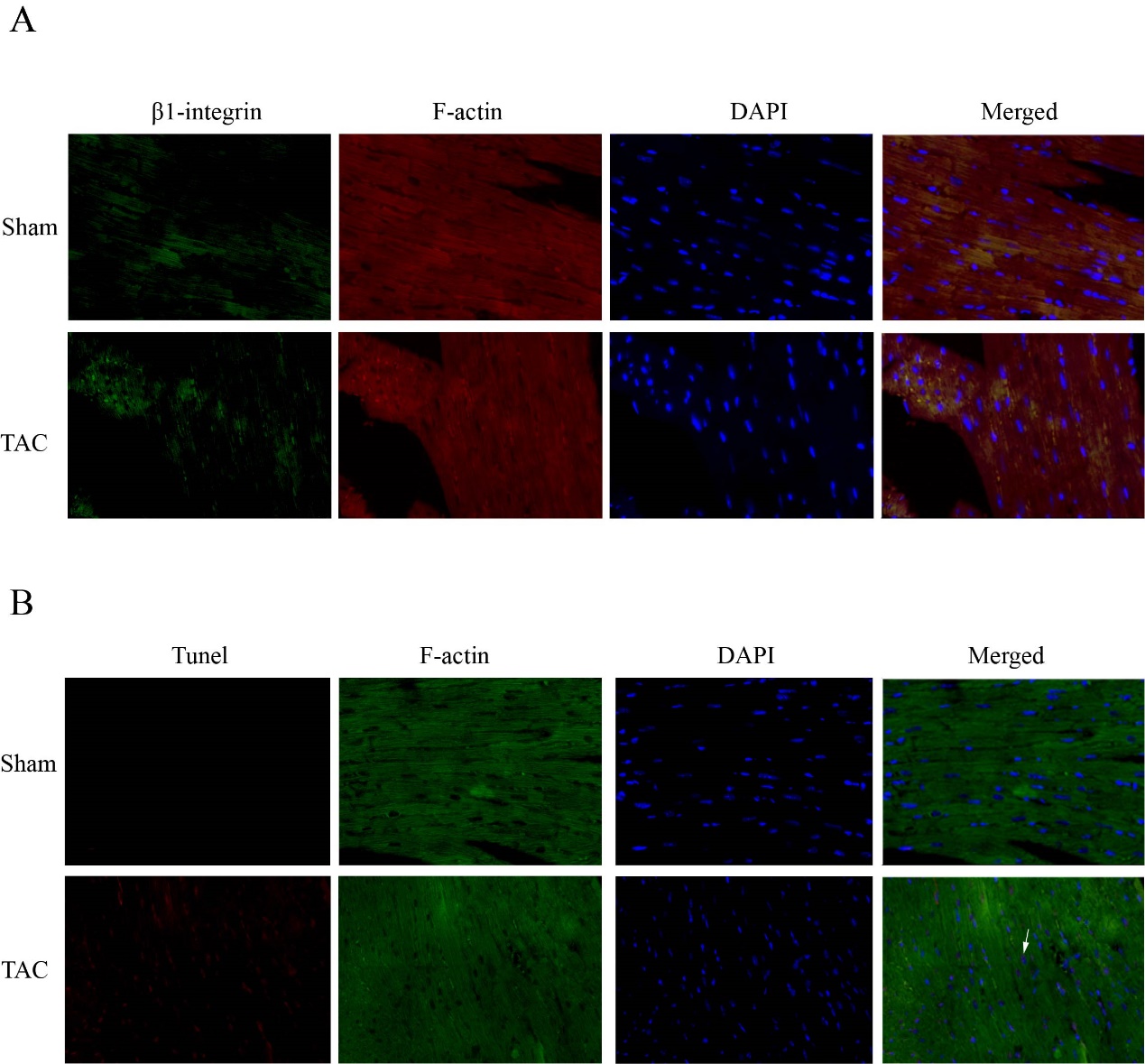


Analysis of the β1 integrin level and apoptosis in cardiomyocytes after 4 weeks of TAC. (A) Immunofluorescence (IF) staining of β1 integrin proteins in cardiomyocytes in the TAC and Sham groups. β1 integrin (Green), F-actin stained with rhodamine phalloidin (red), Nuclei was stained blue with DAPI. Scale bar = 50 μm. (B) TUNEL staining (Red) demonstrated that cardiomyocytes underwent apoptosis in TAC group. F-actin stained with rhodamine phalloidin (Green), Nuclei was stained blue with DAPI. TUNEL-positive nuclei in which TUNEL staining is indicated by arrow. Scale bar = 50 μm.
